# A new species of *Ceratoteleas* Kozlov (Hymenoptera, Scelionidae) from Japan, with a description of the male of *Ceratoteleas*

**DOI:** 10.3897/zookeys.609.8852

**Published:** 2016-08-08

**Authors:** Yoto Komeda, Toshiharu Mita, Kenzo Yamagishi

**Affiliations:** 1 Entomological Laboratory, Graduate School of Bioresource and Bioenvironmental Sciences, Kyushu University, Hakozaki 6–10–1, Fukuoka, 812–8581 Japan; 2 Entomological Laboratory, Faculty of Agriculture, Kyushu University, Hakozaki 6–10–1, Fukuoka, 812–8581 Japan; 3 Entomological Laboratory, Faculty of Agriculture, Meijo University, Shiogamaguchi, Nagoya, 468–8502 Japan

**Keywords:** Taxonomy, Teleasinae, East Asia

## Abstract

*Ceratoteleas cornus* **sp. n.** is described from Japan. The male of the genus is described for the first time.

## Introduction

*Ceratoteleas* Kozlov, 1965 is a monotypic genus belonging to Teleasinae (Scelionidae) (Johnson 1992). The genus is closely related to *Teleas* Latreille (Kozlov 1965), but differs in the presence of the bidentate metanotal spine (sometimes even tridentate in *Teleas*) and the presence of horn on T1 (not always present in *Teleas*). *Ceratoteleas bidentatus* Kozlov 1965, the type species of *Ceratoteleas*, was described from Russian Far East based on 8 females (Kozlov 1965; Kononova and Kozlov 2001). Hereby we represent a new *Ceratoteleas* species based on 42 specimens collected from Honshu Is. and Kyushu Is., Japan.

## Materials and methods

Specimens examined are deposited in the Entomological Laboratory, Kyushu University, Fukuoka. The following abbreviations were used for collecting methods: MT – Malaise trap; YPT – yellow pan trap.

Morphological terminology and measurements mainly follow Mikó et al. (2007, 2010). Postacetabulum is defined as the area on the mesopectus that is delimited anteriorly by the acetabular carina, dorsally by the anterior margin of mesopectus and the mesopleural carina, posteriorly by the ventral mesopleural carina. The description of surface sculpture follows Eady (1968) and Harris (1979), and terms of wing venation follow Masner (1980). Abbreviations used for additional measurements are as follows: A2–6 – length of female antennomere 2–6; A5L – length of male antennomere 5; A5W – apical width of male antennomere 5; ty – length of tyloid in antenomere 5.

## Taxonomy

### Key to species (female)

**Table:** 

1	Gena striate; A3 as long as A2; mesoscutellum with transverse carina; T3 longitudinally striate	***Ceratoteleas bidentatus* Kozlov, 1965**
-	Gena areolate; A3 longer than A2; mesoscutellum without transverse carina; T3 areolate	***Ceratoteleas cornus* sp. n.**

#### Ceratoteleas cornus sp. n.

Taxon classificationAnimaliaHymenopteraScelionidae

http://zoobank.org/5F194C56-510A-44F8-BD8D-54BB4F495A98

##### Description.

***Female*** (*n* = 5): Length = 2.00–2.50 mm (*m* = 2.28, SD = 0.20).

**Figure 1.**
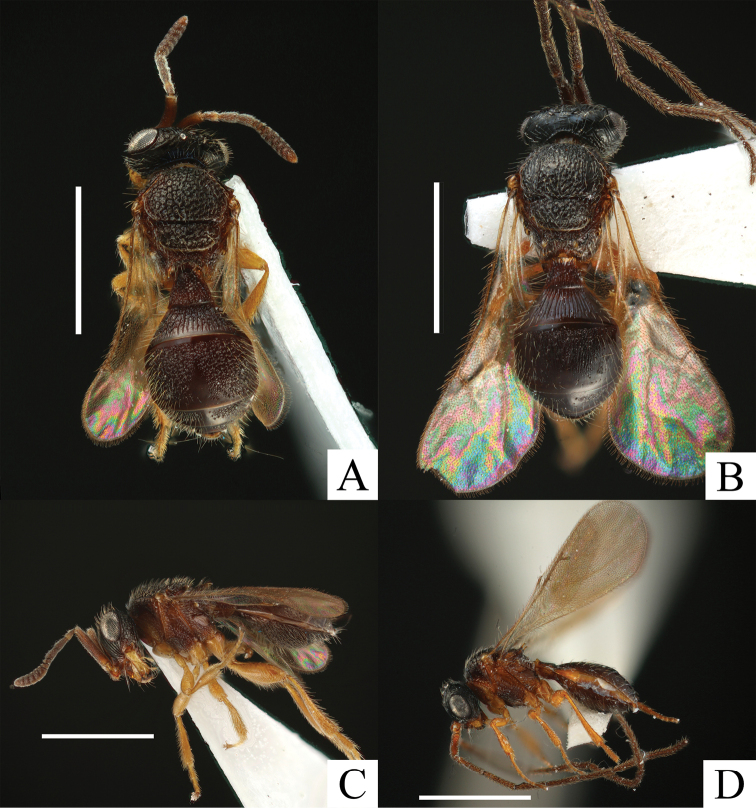
*Ceratoteleas cornus* sp. n. **A** female, dorsal view **B** male, dorsal view **C** female, lateral view **D** male, lateral view. Scale bars = 1 mm.

*Color* (Figs 1A, C). Body dark brown; interantennal process, A1–2, meso- and metapleura (Fig. 1C) brown; radicle, mandible and legs yellow.

**Figure 2.**
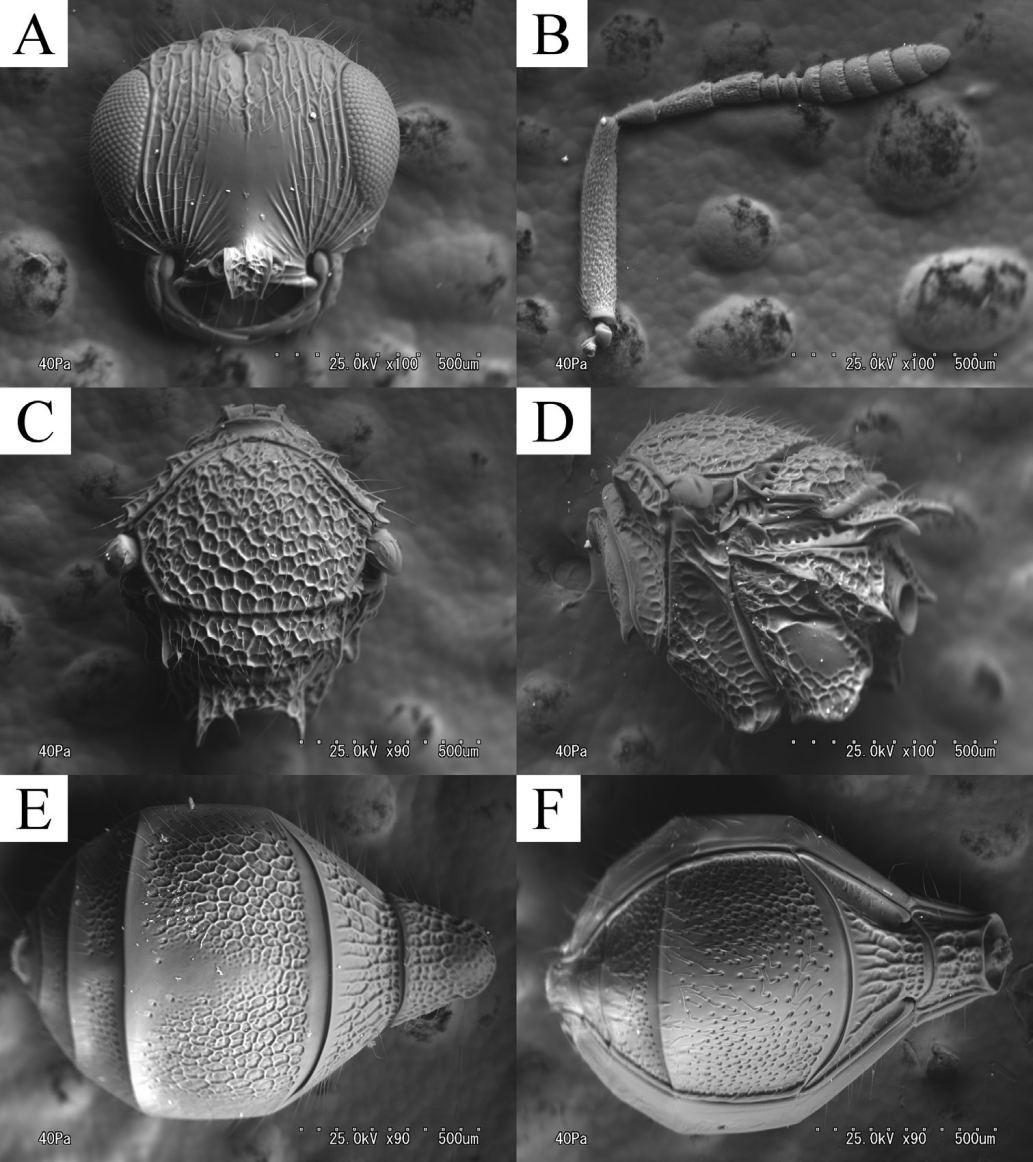
*Ceratoteleas cornus* sp. n., female. **A** head, anterior view **B** antennae **C** mesosoma, dorsal view **D** mesosoma, lateral view **E** metasoma, dorsal view **F** metasoma, ventral view.

*Head*. FCI = 1.27–1.38 (*m* = 1.33, SD = 0.04); LCI = 1.55–1.78 (*m* = 1.61, SD = 0.10); DCI = 2.09–2.30 (*m* = 2.14, SD = 0.09); HW/IOS = 1.50–1.68 (*m* = 1.62, SD = 0.07); head about 1.2 times as wide as mesosoma (HW/TSL = 1.17–1.24, *m* = 1.21, SD = 0.03). Frons (Fig. 2A) dorsoventrally costate with dense long setae; orbital band absent; frontal patch absent; central keel present dorsally; antennal scrobe smooth, without setae; torular triangle smooth; POL as long as OOL (POL/OOL = 0.89–1.00, *m* = 1.00, SD = 0.06); OOL about 2 times as long as LOL (OOL/LOL = 1.80–2.25, *m* = 2.00, SD = 0.18); interantennal process (Fig. 1C) angular, forming right angle ventrally. Vertex costate with dense long setae; interocellar space rugose; hyperoccipital carina absent; vertex patch absent; Eyes with sparse setae. Malar region costate with dense setae; facial striae extending to top of eye; orbital carina present. Gena areolate with dense setae; genal patch absent. A1 (Fig. 2B) about 8 times as long as radicle (A1/r = 7.75–8.75, *m* = 8.25, SD = 0.45), 1.4 times as long as clava (A1/cl = 1.35–1.38, *m* = 1.35, SD = 0.01), about 16 times as long as A6 (A1/A6 = 15.50–17.50, *m* = 16.50, SD = 0.89); A2 3.5 times as long as A6 (A2/A6 = 3.50); A3 longest among A2–6, about 4.5 times as long as A6 (A3/A6 = 4.50–5.00, *m* = 4.50, SD = 0.27); A4 about 3.5 times as long as A6 (A4/A6 = 3.50–4.00, *m* = 3.50, SD = 0.27); A5 as long as A6 (A5/A6 = 1.00). Mandible tridentate; median tooth small, posterior tooth largest.

*Mesosoma*. Pronotal suprahumeral sulcus foveolate with long setae; epomial carina present; cervical pronotal area areolate with dense setae, sculpture finer laterally; lateral pronotal area smooth dorsally, weakly rugulose ventrally. Mesoscutum (Fig. 2C) about 1.4 times as wide as long (TSL/ML = 1.37–1.52, *m* = 1.43, SD= 0.06), areolate with dense long setae; mesoscutal suprahumeral sulcus finely sulcate; mesoscutal humeral sulcus finely sulcate; antero-admedian line absent; notaulus absent. Mesoscutellum (SW/SL = 2.20–2.64, *m* = 2.55, SD= 0.18), areolate with dense long setae; scutoscutellar sulcus present; axillular carina extending posteriorly as a spine; median mesoscutellum without spine; posterior scutellar sulcus present. Femoral depression (Fig. 2D) transversally costate–areolate; mesopleural carina present; postacetabular sulcus present; postacetabulum areolate with dense setae; postacetabular patch absent; sternaulus absent; mesepimeral sulcus weakly foveolate; speculum smooth dorsally, costate ventrally; prespecular sulcus absent. Metanotal trough foveolate; metascutellum areolate, with bidentate spines; metascutellar carina present. Metapleural sulcus deeply impressed; dorsal metapleural area areolate; ventral metapleural area smooth dorsally, areolate laterally and ventrally; paracoxal sulcus absent; metapleural epicoxal sulcus absent; metapleural epicoxal carina absent; metapleural triangle areolate; prespiracular propodeal area modified to tooth; lateral propodeal carina present; lateral propodeal area areolate–rugose; metasomal depression foveolate; plica poorly defined; posterior propodeal projection present; plical area areolate–rugose, with dense setae. Legs (Fig. 1C) robust. Protibia with dense setae; anterior part of protibia with dense spines. Mesotibia with dense setae; anterior part of mesotibia with dense spines. Metafemur swollen; metatibia with dense setae; anteroapical part of metatibia with dense spines. Fore wing (Fig. 1A) extending to apical metasoma, about as wide as mesoscutum (TSL/WW = 1.02–1.30, *m* = 1.18, SD = 0.10); marginal vein about 2.3 times as long as stigmal vein (m/st = 2.17–2.67, *m* = 2.33, SD = 0.19). Hind wing extending to posterior margin of metasoma, about 5.5 times as wide as length of marginal cilia at widest point (HWW/HWS = 5.40–6.25, *m* = 5.50, SD = 0.34).

*Metasoma*. T1 about 0.7 times as wide as T1+T2 length (T1W/T1+T2L = 0.71–0.81, *m* = 0.74, SD = 0.04), areolate, convex anterodorsally, as short horn. T2 areolate; basal depressions on T2 unclear; lateral patch of T2 present, with dense setae. T3 (Fig. 2E) about 1.4 times as wide as long (T3W/T3L = 1.41–1.49, *m* = 1.45, SD = 0.03), about 1.3 times as wide as mesoscutum (T3W/TSL = 1.18–1.29, *m* = 1.28, SD = 0.04), areolate, with dense setae laterally, smooth medially; basal depressions on T3 absent; lateral patch of T3 absent; posterodorsal patch of T3 absent; apical setae on T3 absent. S3 (Fig. 2F) deeply punctate with dense setae; basal depressions on S3 absent. T4 areolate with dense long setae; median patch on T4 absent; lateral patch of T4 absent. T5 areolate with dense long setae; lateral patch of T5 absent. T6 puncutulate with dense long setae; lateral patch of T6 absent.

***Male*** (*n* = 5): Length = 1.88–2.25 mm (*m* = 2.13, SD = 0.15)

*Color* (Fig. 1B, D) similar to female, but lighter.

**Figure 3.**
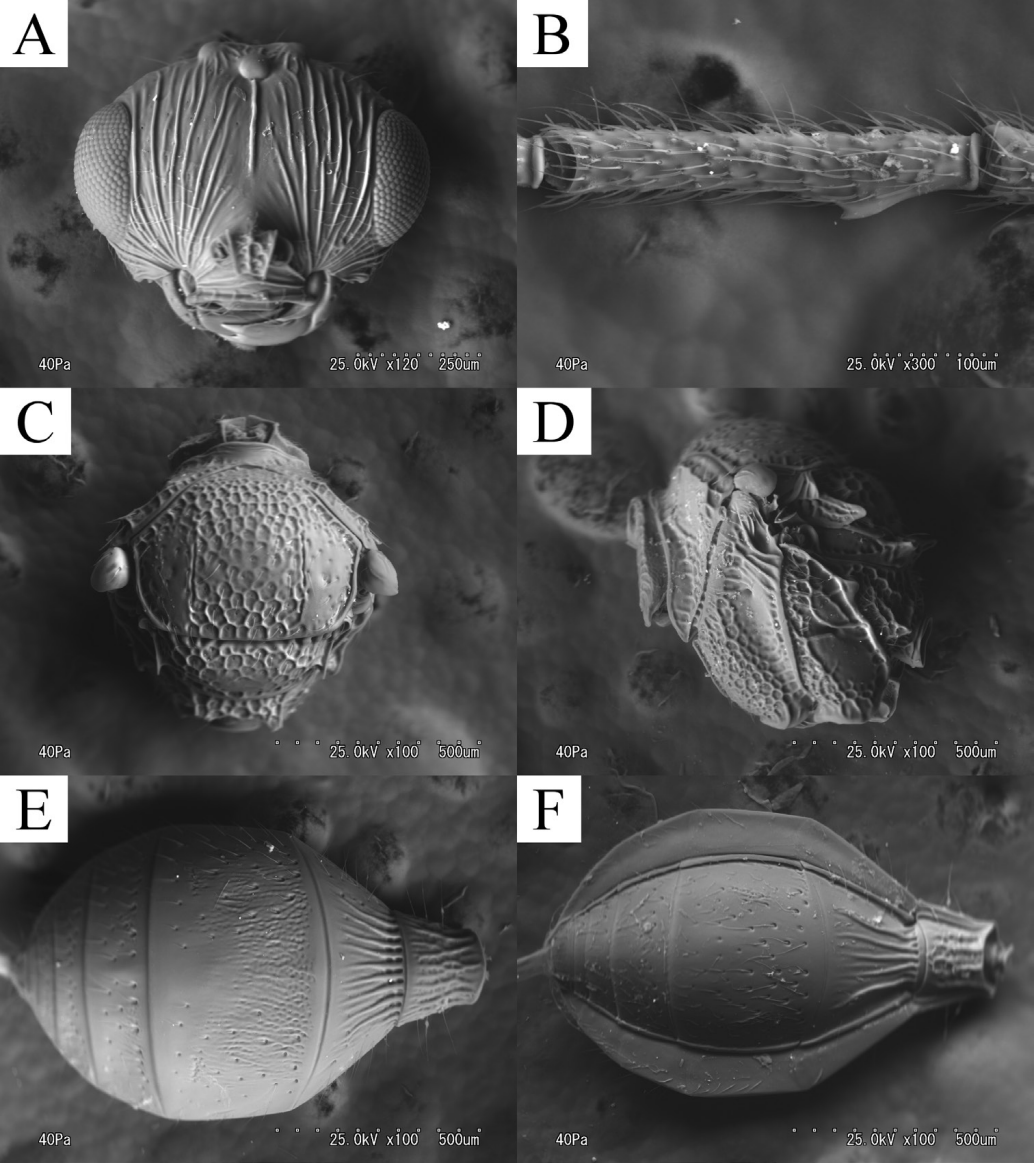
*Ceratoteleas cornus* sp. n., male. **A** head, anterior view **B** antennae **C** mesosoma, dorsal view **D** mesosoma, lateral view **E** metasoma, dorsal view **F** metasoma, ventral view.

*Head*. FCI = 1.37–1.47 (*m* = 1.43, SD = 0.04); LCI = 1.40–1.67 (*m* = 1.50, SD = 0.10); DCI = 2.00 – 2.29 (*m* = 2.15, SD = 0.10); HW/IOS = 1.43–1.47 (*m* = 1.45, SD = 0.02); head about 1.3 times as wide as mesosoma; (HW/TSL = 1.20–1.30, *m* = 1.25, SD = 0.04). Antennal scrobe (Fig. 3A) absent; POL shorter than OOL (POL/OOL = 0.67–0.75, *m* = 0.69, SD = 0.03); OOL about 4 times as long as LOL (OOL/LOL = 3.25–4.00, *m* =4.00, SD = 0.41); interantennal process (Fig. 1D) circular, forming right angle ventrally. Vertex costate–smooth, with dense setae. Clypeus smooth. Gena areolate–costate with dense setae; genal patch absent. A1 about 5.3 times as long as radicle (A1/r = 5.00–5.50, *m* = 5.25, SD = 0.25); A5 (Fig. 3B) about 6.7 times as long as wide (A5L/A5W = 6.33–7.33, *m* = 6.67, SD = 0.38), 3.8 times as long as tyloid (A5L/ty = 3.50–4.00, *m* = 3.80, SD = 0.22).

*Mesosoma*. Cervical pronotal area areolate with dense setae; lateral pronotal area smooth dorsally, areolate ventrally. Mesoscutum (Fig. 3C) about 1.3 times as wide as long (TSL/ML = 1.32–1.43, *m* = 1.33, SD= 0.04), with sparse setae; notaulus weakly present; inter notaular area areolate with sparse setae; lateral notaular area smooth–sparsely punctate. Mesoscutellum about 2.3 times as wide as long (SW/SL = 2.14–2.38, *m* = 2.25, SD= 0.10), areolate with dense setae laterally, smooth dorsally; scutoscutellar sulcus foveolate–sulcate; posterior mesoscutellar sulcus foveolate. Metascutellum shorter than female, with weak bump medially. Metanotal trough (Fig. 3D) foveolate–sulcate. Metasomal depression rugose. Legs (Fig. 1D) slightly robust. Setae and spines of tibiae shorter than female. Fore wing (Fig. 1D) long, exceeding to apical mesosoma, wider than mesoscutellum (TSL/WW = 0.67–0.69, *m* = 0.69, SD = 0.01); marginal vein about 2.8 times as long as stigmal vein (m/st = 2.44–3.00, *m* = 2.78, SD = 0.27). Hind wing long, exceeding to apical mesosoma, about 6 times as wide as length of marginal cilia at widest point (HWW/HWS = 5.60–6.50, *m* = 6.00, SD = 0.32).

*Metasoma*. T1 about 0.4 times as wide as T1+T2 length (T1W/T1+T2L = 0.38–0.54, *m* = 0.41, SD = 0.06), longitudinally costate–areolate. T2 costate; basal depressions on T2 unclear. T3 (Fig. 3E) about 1.5 times as wide as long (T3W/T3L = 1.41–1.59, *m* = 1.50, SD = 0.07), about 1.2 times as wide as mesoscutum (T3W/TSL = 1.13–1.23, *m* = 1.20, SD = 0.04), costate–finely areolate, with sparsely deeply punctate sculpture and setae; lateral patch of T3 present, with dense setae. S3 (Fig. 3F) sparsely deeply punctate with dense setae. T4 costate–finely areolate, with sparsely deeply punctate sculpture and setae; lateral patch of T4 present, with dense setae. T5 areolate, with sparsely deeply punctate sculpture and setae; lateral patch of T5 absent. T6 punculate with dense long setae; lateral patch of T6 absent.

##### Material examined

(See also Suppl. material 2: Specimens data in DarwinCore format.). **Holotype female**: Fukuoka Pref.: Fukuoka city, Mt. Tachibana-yama (prim. evergr. for.), 20. V. – 9. VI. 1979, K. Yamagishi leg. (YPT). **Paratypes**: Hiroshima Pref.: Hatsukaichi city, Yoshiwa vill., Kanmuri Highlands (weed lands), 21. VI. 2015, Y. Komeda leg., 2♂ (YPT); Fukuoka Pref.: Fukuoka city, Mt. Tachibana-yama (Pond), 11. IX. 1993, H. Honda leg., 2♂ (YPT); 18. IX. 1993, 1♂1♀ (YPT); 7. V. 1994, 1♀ (YPT); 12. VI. 1994, 1♂ (YPT); 18. VI. 1994, 7♂3♀ (YPT); 25. VI. 1994, 1♂ (YPT); 2. VII. 1994, 6♂ (YPT); Soeda town, Mt. Hiko-san, 13. V. 1955, T. Esaki, K. Yasumatsu & Y. Hirashima leg. 1♂ (with white determination label; “*Trisacantha* Det. L. Masner, 1974”); 14. VI. 1969, K. Kanmiya leg. 1♂; 31. V. 1971, K. Takeno leg., 1♂; 26. VI – 4. VII. 2008, T. Mita & S. Sato leg., 1♀ (MT); 2–9, VII, 2008, T. Mita & S. Sato leg., 1♀ (MT); Ôita Pref., Kokonoe town, Tano, Chôjabaru, 5–7, V, 2016, T. Mita leg., 11♂ (YPT).

##### Distribution.

Japan (Honshu: Hiroshima; Kyushu: Fukuoka, Ôita).

##### Etymology.

The species name refers to a horn of T1.

## Discussion

Kozlov (1965) established the monotypic genus *Ceratoteleas*, based on *Ceratoteleas bidentatus* Kozlov, 1965. *Ceratoteleas cornus* is the second species of this genus. These two species are similar in areolate mesoscutum and mesoscutellum, and bidantate metanotal spines. However, they can be devided by the sculpture of gena and T3 (striate in *Ceratoteleas bidentatus*; areolate in *Ceratoteleas cornus*), and the ratio of antenomeres lengths (A3 as long as A2 in *Ceratoteleas bidentatus*; A3 longer than A2 in *Ceratoteleas cornus*).

Masner (1976) has recorded *Trisacantha* Ashmead, 1887 from Japan based on a specimen deposited in Entomological Laboratory of Kyushu University. We examined the voucher specimen, and found that the specimen belongs to *Ceratoteleas cornus*. Also, *Trisacantha* is most likely restricted to the Nearctic: in Masner (1980), the distribution of that is only Nearctic. Therefore, Japan is excluded from the distribution of *Trisacantha*.

Diagnostic futures of *Ceratoteleas* are the bidentate metanotal spine (female and male) and the presence of T1 horn (female). Komeda et al. (2015) redescript three species of *Teleas*; *Teleas strigatus* Kozlov, 1965, *Teleas sulcatus* (Kozlov, 1961) and *Teleas tridentatus* (Kozlov, 1961). One of diagnostic futures between male of *Teleas strigatus* and *Teleas sulcatus* is shape of the metanotal spine: triangular (*Teleas strigatus*) or tridentate (*Teleas sulcatus*). Also, that between female of *Teleas strigatus* and *Teleas tridentatus* is absence (*Teleas strigatus*) or weakly presence (*Teleas tridentatus*) of the T1 horn. In addition, Fabritius (1970) synonymized *Proteleas* Kozlov, 1961 because diagnostic futures of it (scutellum with lateral tooth; metanotal spine tridentate; marginal vein 2–2.5 times as long as stigmal; T1 with areolate sculpture) are also common to some species of *Teleas*. Therefore, the shape of metanotal spine and the presence of T1 horn should not regarded as generic diagnosis, but specific. Masner (1976) mentioned another two genera, *Gryonella* Dodd, 1914 and *Echinoteleas* Risbec, 1954, which also have bidentate (*Gryonella*) or tridentate (*Echinoteleas*) metanotal spine. Diagnostic characters of *Teleas* and *Gryonella* are common to *Ceratoteleas. Echinoteleas* differs from *Teleas* in presence of transverse carina on strongly convex mesoscutellum. But Masner (1976) indicated that *Gryonella* might be a synonym of *Teleas*, and *Echinoteleas* might be a synonym of *Teleas* or *Trisacantha* because of molphorogical similarities. Reevaluation of these genera are required to recognize correctly them.

## Supplementary Material

XML Treatment for Ceratoteleas cornus

## Supplementary materials

Supplementary material 1 URI table of HAO morphological terms Data type: specimens data Explanation note: File format: comma-separated values (CSV). This dataset is made available under the Open Database License (http://opendatacommons.org/licenses/odbl/1.0/). The Open Database License (ODbL) is a license agreement intended to allow users to freely share, modify, and use this Dataset while maintaining this same freedom for others, provided that the original source and author(s) are credited.Yoto Komeda, Toshiharu Mita, Kenzo Yamagishi

Supplementary material 2 Specimens data in DarwinCore format Data type: specimens data Explanation note: File format: comma-separated values (CSV). This dataset is made available under the Open Database License (http://opendatacommons.org/licenses/odbl/1.0/). The Open Database License (ODbL) is a license agreement intended to allow users to freely share, modify, and use this Dataset while maintaining this same freedom for others, provided that the original source and author(s) are credited.Yoto Komeda, Toshiharu Mita, Kenzo Yamagishi
